# Safety and biologic activity of a canine anti‐CD20 monoclonal antibody in dogs with diffuse large B‐cell lymphoma

**DOI:** 10.1111/jvim.17080

**Published:** 2024-04-25

**Authors:** Gretchen P. McLinden, Anne C. Avery, Heather L. Gardner, Kelley Hughes, Angie M. Rodday, Kexuan Liang, Cheryl A. London

**Affiliations:** ^1^ Cummings School of Veterinary Medicine Tufts University North Grafton Massachusetts USA; ^2^ College of Veterinary Medicine and Biomedical Sciences Colorado State University Fort Collins Colorado USA; ^3^ Clinical Translational Science Institute Tufts University North Grafton Massachusetts USA

**Keywords:** canine, CD20, lymphoma, monoclonal antibody, veterinary

## Abstract

**Background:**

To explore the safety and utility of combining low dose single‐agent doxorubicin with a canine specific anti‐CD20 monoclonal antibody (1E4‐cIgGB) in client owned dogs with untreated B‐cell lymphoma.

**Animals:**

Forty‐two client‐owned dogs with untreated B‐cell lymphoma.

**Methods:**

A prospective, single arm, open label clinical trial of dogs with B‐cell lymphoma were enrolled to receive 1E4‐cIgGB and doxorubicin in addition to 1 of 3 immunomodulatory regimens. B‐cell depletion was monitored by flow cytometry performed on peripheral blood samples at each visit.

**Results:**

Dogs demonstrated a statistically significant depletion in CD21+ B‐cells 7 days following the first antibody infusion (median fraction of baseline at 7 days = 0.04, *P <* .01) that persisted throughout treatment (median fraction of baseline at 21 days = 0.01, *P* < .01) whereas CD5+ T‐cells remained unchanged (median fraction of baseline at 7 days = 1.05, *P* = .88; median fraction of baselie at 7 days = 0.79, *P* = .42; Figure 1; Supplemental Table 3). Recovery of B‐cells was delayed, with at Day 196, only 6/17 dogs (35%) remaining on the study had CD21+ counts >0.5 of baseline, indicating sustained B cell depletion at 4+ months after the final treatment. 1E4‐cIgGB was well tolerated with only 1 dog exhibiting a hypersensitivity event within minutes of the last antibody infusion.

**Conclusions:**

The canine 1E4‐cIgGB anti‐CD20 monoclonal antibody is apparently safe when administered with doxorubicin and effectively depletes B‐cells in dogs with DLBCL.

**Abbreviations**
ADCC: antibody dependent cell‐mediated cytotoxicity
AEs: adverse events
CR: complete remission
DLBCL: diffuse large B‐cell lymphoma
MST: median survival time
ORR: objective response rate
OST: overall survival time
PFS: progression free survival
PR: partial remission
SD: stable disease

## 1 INTRODUCTION

Lymphoma occurs frequently in dogs, representing 83% of all hematopoietic neoplasia and 7%‐24% of all malignancies in dogs.^1^, ^2^, ^3^, ^4^ Treatment of B‐cell lymphoma, the most common subtype in dogs, involves multi‐agent chemotherapy typically with CHOP‐based protocols incorporating cyclophosphamide, doxorubicin, vincristine, and prednisone, with more than 80% achieving a complete remission (CR). The median survival time (MST) for dogs with B‐cell lymphoma treated with CHOP ranges from 10 to 14 months and includes the use of rescue therapies after relapse.^5^, ^6^, ^7^ Importantly, no clinically important improvements in MST for dogs with B‐cell lymphoma treated with chemotherapy have occurred over the past 3 decades.

In human patients, diffuse large B‐cell lymphoma (DLBCL) is treated with CHOP‐based chemotherapy in combination with rituximab, a humanized monoclonal antibody that targets CD20 expressed on the surface of both normal and neoplastic B‐cells (R‐CHOP).^8^ Rituximab contributes to the depletion of B‐cells through several mechanisms including complement and antibody dependent cell‐mediated cytotoxicity (ADCC).^9^, ^10^, ^11^, ^12^ Historically, the use of CHOP alone was associated with a 5‐year survivorship of approximately 45%.^13^ With the addition of rituximab to CHOP, the overall 5‐year survivorship has improved to 78.5%,^13^, ^14^, ^15^ with 70% of patients alive at 10 years.^16^ However, several factors influence outcome including tumor genomics, age, performance status and co‐morbidities, among others, resulting in a range of survival at 10 years from 30% to 90%.^16^

As is the case in humans, both mature and neoplastic canine B‐cells express CD20,^17^ supporting the notion that targeting this cell surface antigen would have similar efficacy in dogs with B‐cell lymphoma. Ritixumab does not bind canine CD20 and does not deplete canine B cells^18^ and its use would be limited in dogs given the likely rapid generation of neutralizing antibodies as has been observed when humanized antibodies are used in dogs.^19^ To address this gap, several groups have endeavored to generate a canine‐specific anti‐CD20 antibody with some degree of “caninization.” The Genomics Institute of the Novartis Research Foundation and Elanco Animal Health collaborated to develop a rituximab‐like canine chimeric anti‐CD20 antibody (1E4‐cIgGB) that demonstrated effective dose dependent and durable depletion of B‐cells in healthy beagle dogs.^20^ Another caninized anti‐CD20 antibody (Blontress, Aratana Therapeutics), received conditional licensure from the USDA. However, B‐cell depletion was never demonstrated in healthy dogs and no published data regarding efficacy in dogs with B cell lymphoma were published. Additional groups have since generated canine specific anti‐CD20 antibodies including 1 that showed high‐affinity binding to CD20 on normal and neoplastic canine B‐cells and promoted phagocytosis of lymphoma cells by macrophages, but did not induce direct or complement dependent cytotoxicity.^21^ Another group developed a recombinant anti‐CD20 single chain (scFv) antibody that bound both human and canine CD20 in vitro.^22^ More recently, another canine specific anti‐CD20 antibody, 4E1‐7‐B, was developed that, similar to 1E4‐cIgGB, induced substantial B‐cell depletion in healthy beagle dogs.^23^ Together these data indicate that targeting anti‐CD20 in dogs with DLBCL represents a viable therapeutic strategy.

As part of a funded National Cancer Institute (NCI) Cancer Moonshot award (U01 CA224153), clinical studies were initiated in pet dogs with DLBCL to explore the utility of combining low dose single‐agent chemotherapy (doxorubicin) with unique immune‐modulatory agents, including 1E4‐cIgGB, that maintained efficacy whereas reducing adverse events associated with dose intense chemotherapy. The purpose of this study was to evaluate the efficiency of B‐cell depletion and adverse event profile associated with administration of 1E4‐cIgGB in dogs with DLBCL treated as part of this NCI funded work.

## 2 MATERIALS AND METHODS

### 2.1 Study animals and inclusion/exclusion criteria

This was an open label non‐randomized clinical trial conducted in dogs with previously untreated DLBCL. Dogs were enrolled if they had a diagnosis of CD21+/CD5− lymphocytosis/B‐cell lymphoproliferative disease on flow cytometric evaluation of lymph node fine needle aspirate samples and confirmed as B‐cell lymphoma on lymph node biopsy, were at least 1 year of age and had a body weight of 8 kg or higher. Dogs must have had at least 2 peripheral lymph nodes that measured ≥2 cm in diameter and adequate organ function as indicated by standard laboratory tests (complete blood count, serum biochemistry profile, urinalysis). Informed written consent from the dog owners was obtained before enrollment. Dogs were excluded if they were pregnant or lactating, had evidence of central nervous system involvement, any autoimmune disease, evidence of underlying cardiovascular/renal/hepatic disease that may impact survival or tolerability of doxorubicin chemotherapy, were less than 2 weeks from a major surgical procedure, or were receiving medications that would confound the interpretation of adverse events or antitumor activity associated with the study, including steroid use before the start of treatment. The animal protocol associated with this study was approved by the Tufts University Institutional Animal Care and Use Committee (IACUC, #G2017‐110, #G2020‐82).

### 2.2 Study design

At the time of screening, dogs underwent physical exam, baseline complete blood count with clinical pathology review, chemistry panel, urinalysis, thoracic radiographs, lymph node aspiration cytology, and lymph node biopsy. A peripheral blood sample and lymph node aspiration sample were submitted to the Clinical Hemopathology Laboratory at Colorado State University for flow cytometric evaluation. Immunophenotyping results were confirmed by Dr. Anne Avery (Director, Clinical Hematopathology Laboratory, Colorado State University). The lymph node biopsy samples underwent histopathologic evaluation and CD3/CD20 immunostaining at the Colorado State University Veterinary Diagnostic Laboratory and were read by a single pathologist to confirm diagnosis (Dr. Kelly Hughes).

### 2.3 Drug products and administration

All dogs received 1E4‐cIgGB, the canine chimeric monoclonal anti‐CD20 antibody,^20^ along with varying combinations of doxorubicin and immunomodulatory study medications defined by the study cohort (Table 1) and detailed in a previous publication.^24^ The antibody was supplied by Elanco Animal Health as a 30.7 mg/mL solution and stored at 4°C. Before administration, dogs were administered diphenhydramine (2 mg/kg SC) and dexamethasone sodium phosphate (0.2 mg/kg IV) to prevent possible infusion related reactions as these are a well‐established complication associated with rituximab administration and a similar premedication strategy is employed in humans.^25^ The antibody was diluted to 4 mg/mL in 0.9% NaCl before IV administration over 90 minutes. All dogs received a loading dose of 1E4‐cIgGB at 20 mg/kg for the first treatment and 10 mg/kg for subsequent treatments based on the earlier healthy dog study demonstrating efficient depletion at 5‐30 mg/kg and limitations associated with a fixed quantity of 1E4‐cIgGB available for the study.^20^ KPT‐9274 (Karyopharm Therapeutics, Newton, USA) was supplied as capsules filled with API consisting of 5, 20, and 50 mg and administered at a dose of 4‐4.5 mg/kg orally once after doxorubicin administration.^26^ TAK‐981 (Takeda Pharmaceuticals, Tokyo, Japan) was supplied as a 10 mg/mL solution and stored at −20°C and given at a dose of 2.5‐3 mg/kg.^27^, ^28^, ^29^, ^30^ RV1001 (Rhizen Pharmaceuticals AG, Basel, Switzerland) was supplied as tablets consisting of 25, 100, 250, and 400 mg active ingredient and was administered orally at a dose of 10‐15 mg/kg.^31^, ^32^ The TAK‐981 was diluted in 0.9% NaCl to 1.8‐2.0 mg/mL and administered over 30 minutes. Additional information regarding immunomodulatory study medications used in this study and their mechanisms of action is provided in Table S1 and is detailed elsewhere.^24^ Doxorubicin was supplied as a 2 mg/mL solution and was administered at a dose of 20‐25 mg/m^2^, or 0.8‐0.9 mg/kg for dogs 10 kg or less. The dose was set intentionally low as part of the chemotherapy‐light regimen designed to mitigate cytotoxic drug toxicosis by leveraging unique combinations of immunomodulatory agents.

**TABLE 1:** Treatment protocols.

Cohort	Treatment protocol
2	1E4‐cIgGB: (20 mg/kg for the first dose, then 10 mg/kg) q3w ×4 Doxorubicin: 20‐25 mg/m^2^ IV q3w ×4 KPT‐9274: 4‐4.5 mg/kg PO with doxorubicin
3	Doxorubicin: 20‐25 mg/m^2^ IV once on Day 0 1E4‐cIgGB (20 mg/kg for the first dose, then 10 mg/kg) q1w ×4 starting on Day 7 TAK‐981: 2.5‐3 mg/kg IV q1w ×4, starting on Day 7
4	1E4‐cIgGB: (20 mg/kg for the first dose, then 10 mg/kg) q3w ×4 Doxorubicin: 20‐25 mg/m^2^ IV q3w ×4 RV1001: 7.5‐15 mg/kg PO 4 days on/3 days off for 3 months
5	1E4‐cIgGB: (20 mg/kg for the first dose, then 10 mg/kg) q3w ×4 Doxorubicin: 20‐25 mg/m^2^ IV q3w ×4 KPT‐9274:4‐4.5 mg/kg PO with doxorubicin RV1001: 7.5‐15 mg/kg PO 4 days on/3 days off for 6 months
6	1E4‐cIgGB: 10‐20 mg/kg IV (20 mg/kg for the first dose, then 10 mg/kg) Doxorubicin: 20 mg/m^2^ IV TAK‐981: 2.5‐3 mg/kg IV Three cycles of: Day 0: 1E4‐cIgGB/Doxorubicin/TAK‐981 Day 7: 1E4‐cIgGB/TAK‐981 Day 14: No treatment

### 2.4 Concomitant medications

Concomitant medications used included: metoclopramide, ondansetron, maropitant, famotidine, omeprazole, metronidazole, diphenhydramine, tramadol, dexamethasone, and prednisone (only permitted if used to treat a transfusion reaction or transaminitis secondary to RV1001 administration). Some dogs had signs of anxiety or other behavior abnormalities that resulted in the use of a pre‐treatment sedation protocol typically consisting of the trazodone, gabapentin, melatonin, transmucosal acepromazine, or a combination of these medications.^33^ This was instituted in consultation with the Tufts Cummings School Behavior Service to reduce stress associated with the sample‐intensive study visits and to facilitate administration of the IV infusions that were typically 2 hours in duration.

### 2.5 Flow cytometry

Flow cytometry of blood and lymph node aspirates were performed using methods and antibody combinations, clones and sources as previously described.^5^, ^34^ This was performed by the Clinical Hematopathology Laboratory at the College of Veterinary Medicine and Biomedical Sciences at Colorado State University. All results were analyzed by Dr. Anne Avery.

### 2.6 B‐cell and T‐cell quantification and depletion

B‐ and T‐cell counts were determined by drawing a gate around the entire leukocyte population plotted as linear forward scatter and log side scatter, determining the percentage of CD21+/CD5− B‐cells or CD5+/CD21− T‐cells (respectively), and then multiplying that percentage by the total white blood cell count. Depletion was assessed as a fraction of the baseline for each individual dog rather than by absolute numbers as the baseline values varied widely. This fraction was calculated by dividing the absolute count at baseline by the absolute count at each time point. For example, for the first dog enrolled, the total CD21+ cell count at D0 (baseline) was 327.1/μL (fraction of baseline = 1). On Day 7, CD21+ cell count was 18/μL (fraction of baseline = 0.055). The same approach was used to assess parallel changes in T‐cell counts throughout the course of treatment.

### 2.7 Response and toxicity criteria

Response to treatment was determined through peripheral lymph node caliper measurements after published guidelines (Response Evaluation Criteria for Peripheral Nodal Lymphoma in Dogs v1.0).^35^ Dogs were assessed weekly for the first 3‐4 weeks, depending upon the protocol used, and then once every 3 weeks for a total of 12 weeks after which dogs were evaluated once monthly until disease progression was noted. Each study visit included physical exam, CBC with clinical pathology evaluation, chemistry, lymph node measurement, collection of blood for flow cytometric analysis and determination of adverse events (AEs). The AEs were defined and graded according to the previously established VCOG‐CTCAE criteria.^36^

### 2.8 Statistical analysis

Recovery was defined as a CD21+ fraction of baseline score of 0.5 at Day 196 (representing a 50% recovery to the baseline score). CD21+ and CD5+ fraction of baseline scores were reported by response (CR vs partial remission, PR) and completion of protocol treatment. To account for dropout over time, separate analyses were done to assess early and late response, by restricting the sample analysis to dogs with complete data through Day 21 and Day 196, respectively. Summary statistics and boxplots were used to describe CD21+ and CD5+ fraction of baseline scores over time, with a focus on medians and quartiles because of the skewed distribution of CD21+ cells. To assess early response, median Day 7 and Day 21 CD21+ and CD5+ fraction of baseline scores were compared to 1 (which would indicate a return to baseline values) using the Wilcoxon signed rank test. To assess later response, Day 7, 21, 84, 112, and 196 CD21+ and CD5+ fraction of baseline scores were compared to 1 (which would indicate a return to baseline values) using the Wilcoxon signed rank test. Data were analyzed using SAS Enterprise Guide version 7.12 (SAS Institute Inc., Cary NC, USA) and R (version 4.2.1)/RStudio (version 2022.07.2). A 2‐sided alpha of 0.05 was used.

## 3 RESULTS

### 3.1 Study demographics

Forty‐two client owned dogs with previously untreated multicentric B‐cell lymphoma were enrolled in this study (Table 2). All dogs had confirmation of B‐cell immunophenotype as assessed by CD21 expression on the malignant lymphocytes collected through fine needle aspiration of an affected lymph node. Of these, 37 dogs had a histopathologic diagnosis of DLBCL, 2 had a histopathologic diagnosis of small to intermediate B‐cell lymphoma, and 1 dog had a histopathologic diagnosis of intermediate B‐cell lymphoma. Two cases did not have a histopathologic diagnosis: 1 sample was non‐diagnostic, and 1 sample was lost in transit to the diagnostic laboratory. However, flow cytometry of fine needle aspiration samples was consistent with DLBCL in both cases, and as such these dogs were included in the trial.^5^, ^37^ The majority (n = 16, 38%) of dogs were classified as mixed breed (either as “mix” or a “breed mix”). Five (12%) were pit bull terriers, 2 of each were beagles, German shepherds, golden retrievers, Labrador retrievers and there was 1 of each for Basset hound, bulldog, cattle dog, cocker spaniel, mastiff, pug, rottweiler, schnauzer, Scottish terrier, Shetland sheepdog, Shiba inu, Wheaton terrier, and Yorkshire terrier. There were 19 females (2 intact, 17 spayed) and 23 males (1 intact, 22 castrated). The median age of dogs enrolled in this study was 7.5 years (range, 2‐13 years).

**TABLE 2:** Demographics.

Age (years)	
Mean	7.5
Median	7.5
Range	2‐13
Breed	
Basset hound	1
Beagle	2
Bulldog	1
Cattle dog	1
Cocker spaniel	1
German shepherd	2
Golden retriever	2
Labrador retriever	2
Mastiff	1
Mix	16
Pitbull	5
Pug	1
Rottweiler	1
Schnauzer	1
Scottish terrier	1
Shetland sheepdog	1
Shiba inu	1
Wheaton terrier	1
Yorkshire terrier	1
Gender	
Female	2
Spayed female	17
Male	1
Castrated male	22
Stage	
IIIa	26
IIIb	1
IVa	7
IVb	2
Va	6

### 3.2 Clinical stage

Staging was performed through physical examination, CBC with clinical pathology evaluation, chemistry profile, thoracic radiographs (40/42 cases) and flow cytometry of the peripheral blood to identify circulating neoplastic cells. 7/42 dogs underwent abdominal ultrasonographic examination as part of screening, and as such, involvement of the spleen and liver was typically assessed through abdominal palpation to identify cranial organomegaly. Of the 42 dogs enrolled, 27 were deemed to be stage III (n = 26 substage a, n = 1 substage b) and 9 stage IV (n = 7 substage a, n = 2 substage b) at the time of enrollment. An additional 6 dogs were classified as stage Va (no substage b) based on large mononuclear cells identified on the pretreatment CBC concerning for circulating lymphoblasts, that were subsequently confirmed to be lymphoma by flow cytometry.

### 3.3 Overall response to treatment

A total of 29 (69% of) dogs completed the designated protocol and received all intended doses of drug. Once dogs developed progressive disease (PD) and were officially off study, they were followed for overall survival, but serial blood collections to assess circulating B‐ and T‐cell populations were no longer undertaken. Therefore, number of dogs actively followed during the study decreased over time secondary to loss from PD. With respect to treatment response, most dogs (n = 33, 79%) experienced a CR by 21‐28 days after treatment initiation, with an additional 9 (21%) experiencing a PR, for an overall response rate (ORR) of 100% (see Table S2 for response by cohort). However, none of the dogs that had experienced a PR went on to complete the entire assigned treatment protocol.

### 3.4 B‐cell depletion and recovery

**FIGURE 1:**
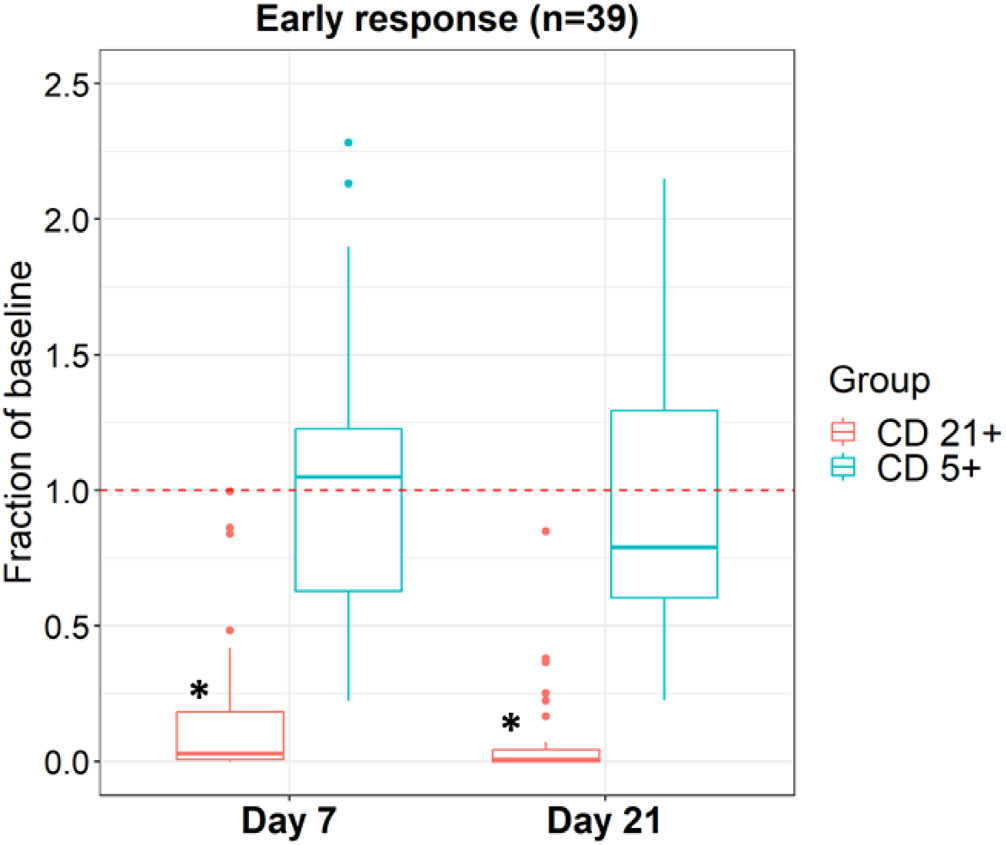
Dynamics of early B cell depletion. Box plot for dogs still on study at d21 (n = 39) demonstrating fraction of baseline of CD21+/CD5− cells (red) and CD5+/CD21− cells (blue). CD21+ cells and CD5+ cells quantified as a fraction of baseline at d7 and then d21. CD21+ fraction of baseline was significantly reduced at both time points (*P < .01) whereas CD5+ fraction of baseline had no statistical difference (*P* = .88 at d7, *P* = .42 at d21).

As shown in Figure 1, CD21+ B‐cells were significantly decreased by Day 7 after the first dose of 1E4‐cIgGB and this persisted through Day 21 (*P* < .01 at Day 7 and Day 21). CD5+ T‐cells remained unchanged at both time points (*P* = .88 at Day 7 and *P* = .42 at Day 21). Of the 17 dogs that remained in remission and on study at Day 196, 11 (65%) exhibited persistent CD21+ B‐cell depletion more than 4 months after the last infusion of IE4‐cIgGB (Table S3).

In 3 dogs, 1E4‐cIgGB had minimal effect on CD21+ B‐cell numbers after the first infusion. Two of these dogs continued to exhibit fluctuations in B‐cell numbers throughout the study that did not correlate with physical exam findings as they were deemed to be in a CR. The first case demonstrated B‐cell depletion fractions ranging from 0.379 at Day 21 to 4.803 at Day 196. Despite these variations, the dog remained in a CR until PD was confirmed at Day 297. The second dog had B‐cell depletion fractions ranging from 0.849 at Day 21 to 7.15 at Day 196. This dog was still in a CR at the time of publication. The third dog demonstrated only partial B‐cell depletion 0.485 at Day 7 and 0.367 at Day 21 at which point PD resulted in removal from the study. All 3 of these dogs had confirmed DLBCL diagnosed via lymph node biopsy before starting treatment.

**FIGURE 2:**
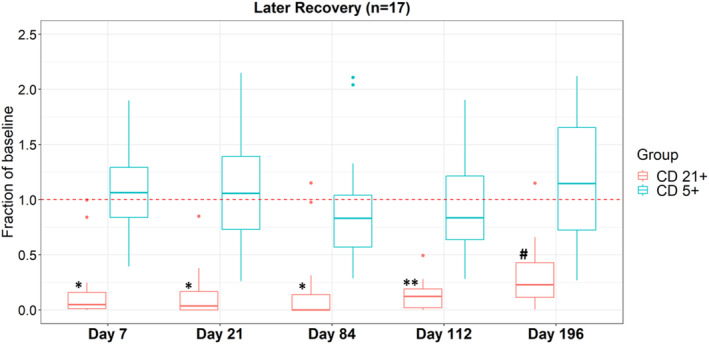
Timeline of B cell recovery. Box plot for dogs still on study at d196 (n = 17) demonstrating fraction of baseline of CD21+/CD5− cells (red) and CD5+/CD21− cells (blue). Statistically significant decrease in fraction of baseline seen in CD21+ cells at D7, D21, D84 (*P < .01), and D12 (**P = .04) but not at d196 (^#^ *P* = .21). There was no statistical differences in CD5+ cells across all time points analyzed (*P* = .40 at d7, *P* = .71 at d21, *P* = .21 at d84, *P* = .58 at d112, *P* = .26 at d196).

B‐cell recovery was typically delayed after completion of treatment, often by several months. For example, of the 17 dogs that completed the protocol with data points extending to at least Day 196, 6 (35%) had CD21+ counts that were >0.5 of baseline at Day 196, representing a statistically insignificant degree of depletion (*P* = .21; Figure 2). To better characterize how long B‐cell recovery took, CD21+ counts were evaluated for dogs still on study (ie, remained in a CR) at Day 300. Of the 6 dogs in this group, 4 (66.7%) had CD21+ counts that were >0.5 of baseline confirming that recovery of B cells was significantly delayed in some dogs.

The extent of B‐cell depletion noted in dogs at Day 7 or 21 was not predictive of overall outcome in the current study. There was also no difference in CD21+ or CD5+ cell counts at Day 7 between dogs with a CR vs those with a PR (Table S4). Lastly, CD21+ or CD5+ cell counts at Day 7 or Day 21 were not predictive of protocol completion (Table S5). Therefore, effective depletion of normal B‐cells in the peripheral blood was not a surrogate biomarker associated with overall outcome. In contrast, 10 dogs (23.8%) developed increases in CD21+ B‐cell counts that coincided with relapse, possibly representing emergence of circulating lymphoma cells in these cases.

### 3.5 Adverse events

A total of 160 doses of 1E4‐cIgGB were administered to dogs in this study, with a median of 4 doses given (range, 1‐6). One dog had an acute vomiting episode during the 1E4‐cIgGB infusion that resolved with an injection of maropitant and lowering the rate of infusion. This same dog then had an episode of suspected Type I hypersensitivity reaction (anaphylaxis) immediately after the last antibody infusion (dose 6/6) that rapidly responded to the administration of steroids, fluids, and epinephrine. No additional infusion related reactions or hypersensitivity events were determined to be directly associated with the 1E4‐cIgGB administration. Five dogs developed grade 1 or 2 azotemia, and these were treated as suspected pyelonephritis as despite negative urine cultures, renal values generally improved with empirical antibiotic therapy (Table 3). In people, rituximab mediated depletion of normal B‐cell populations is associated with a higher risk for bacterial infection, including cystitis and pyelonephritis.^38^ Gastrointestinal and hematologic adverse events were noted throughout study, but all were consistent with toxicities commonly observed with the other agents included in the various treatment regimens (doxorubicin, RV1001, TAK‐981). No dogs were hospitalized for gastrointestinal or hematologic adverse events.

**TABLE 3:** Adverse events.

Adverse event	Grade 1	Grade 2	Grade 3	Grade 4
Nausea	8	1		
Vomiting	8	3		
Diarrhea	10	5		
Lethargy	12	6		
Arrhythmia		2		
Cough	2			
Aspiration pneumonia		1		
Increased respiratory effort	1			
Allergic reaction/hypersensitivity event		1	1	
Pyoderma		4		
Dry skin	1			
Neutropenia	5	6	4	
Thrombocytopenia	2	4	1	
Elevated BUN	1	4		
Elevated Creatinine	4	1		
Elevated total bilirubin	1			1
Elevated ALT	3	1	1	1

## 4 DISCUSSION

While canine DLBCL responds well to multi‐agent CHOP‐based chemotherapy with over 80% of dogs achieving CR, the MST for affected dogs has remained at approximately 1 year for over 3 decades.^5^, ^6^, ^39^ Treatment of human DLBCL has benefited from the addition of the anti‐CD20 monoclonal antibody rituximab, and R‐CHOP is now considered the standard of care.^8^, ^40^, ^41^ As previously discussed, several canine specific CD20 targeted therapeutics have been reported, although only 2 of these have been shown to deplete circulating B cells in healthy dogs.^21^, ^22^ While another purported caninized anti‐CD20 antibody, Blontress (AT‐004, Aratana Therapeutics) was conditionally licensed by the USDA in 2015 to aid in the treatment of canine B‐cell lymphoma, binding of this product to canine CD20 and depletion of canine B cells in vivo were never demonstrated. A different approach was taken by Merial/ Boehringer Ingelheim, which created a xenogenic murine CD20 DNA therapeutic vaccine for use in dogs with B‐cell lymphoma. While clinical trials were initiated, no peer‐reviewed data has been published regarding efficacy and the vaccine is no longer available. Consequently, the study reported here represents the first evaluation of a canine specific anti‐CD20 monoclonal antibody (1E4‐cIgGB) in dogs with DLBCL.

Consistent with the actions of rituximab in humans, our data demonstrates that dogs treated with 1E4‐cIgGB exhibit durable depletion of circulating B‐cells, mirroring published data regarding 1E4‐cIgGB activity in healthy research dogs.^20^ In humans, rituximab mediated B‐cell depletion typically persists for 6‐9 months after completion of R‐CHOP, with recovery to pre‐treatment levels by 12 months.^42^, ^43^ Interestingly, while memory B‐cell responses to earlier vaccine antigen (tetanus toxin) were markedly diminished, hemagglutinin‐specific IgG‐secreting B cell responses could be stimulated by influenza vaccination in patients with DLBCL that had peripheral B‐cell depletion after completion of R‐CHOP.^42^ In healthy research dogs treated with 1E4‐cIgGB, at 81 days post‐injection (the latest time point evaluated), circulating B‐cell populations remained depleted at 41%‐48% of baseline.^4^ In our study, only 35% (6/17) dogs had CD21+ counts >50% of baseline at Day 196 of the study, which represented 4‐5 months post last dose of 1E4‐cIgGB, depending on cohort. While only 6 dogs remained in CR at Day 300, 4 of these (66.7%) had recovered CD21+ counts to >50% of baseline, suggesting that dynamics of B‐cell recovery in both species are is similar.

In human patients with DLBCL treated with R‐CHOP, depletion of B‐cells has been documented as a relevant biomarker associated with response to treatment.^42^ In our study, completeness of peripheral B‐cell depletion did not always correlate with response to treatment. Additionally, it did not predict overall outcome. It is likely that the small sample size per cohort and use of several different immunomodulatory drugs in combination made it challenging to explicitly link the use of 1E4‐cIgGB to progression free survival (PFS) and OST. In support of this, we have recently demonstrated that baseline tumor gene expression was linked to response and outcome to the chemoimmunotherapy protocols used to treat dogs with DLBCL in this study.^24^ Specific associations included increased expression of *CREBBP* and *CDKN1A* for response to KPT‐9274, increased *TLR3* for response to TAK‐981, and increased *PI3Kδ*, *AKT3*, and *PTEN*, and decreased *NRAS* for response to RV1001.^24^ Future controlled prospective studies that include a larger sample size and incorporate a fixed chemoimmunotherapy regimen would help elucidate the impact of 1E4‐cIgGB and associated B‐cell depletion on outcomes.

While it is possible that doxorubicin contributed to the B‐cell depletion noted after 1E4‐cIgGB treatment, a previous study evaluated B‐ and T‐ cell counts in dogs with lymphoma and found no significant changes dogs treated with doxorubicin, but a significant decrease in B‐cells for those receiving CHOP.^44^ Given this finding, and the fact that T‐cell numbers were not affected by treatment, it is most likely that the B‐cell depletion noted in our study population was a direct result from the 1E4‐cIgGB. This is further supported by the data in healthy beagle dogs demonstrating specific targeting of B‐cells after antibody administration,^20^ and by the prolonged recovery noted in dogs that remained on study months after the last doxorubicin dose. It is also possible that B‐cell depletion could have been influenced by 1 of the immunotherapy agents used in this trial. KPT‐9274 is a small molecule NAMPT/PAK4 inhibitor that was given orally with the doxorubicin chemotherapy and was not continued between treatments. Given its short half‐life and mechanism of action, it is unlikely that the KPT‐9274 affected the normal B‐cell population.^45^, ^46^ In mouse models, the SUMO‐activating enzyme inhibitor TAK‐981 did induce transient decreases in normal B‐cell populations in the spleens and blood of mice, but these rapidly recovered within 4 days after discontinuation of treatment.^47^ It is therefore conceivable that the TAK‐981 acted in an additive or synergistic manner with 1E4‐cIgGB to selectively deplete B‐cells. Last, while PI3Kδ inhibition does interfere with B‐cell receptor (BCR) signaling, it does not deplete normal B‐cells and “tonic signaling” of the BCR can occur via either PI3Kα or PI3Kδ. Moreover, mice deficient in PI3Kδ still develop B‐cells.^48^ Therefore, despite the possible contributions of both RV1001 and TAK‐981 to B‐cell depletion, we did not observe differences in the extent and duration of B‐cell loss among the treatment groups and thus could largely attribute the observed effect to 1E4‐cIgGB.

The anti‐CD20 antibody used in this study was well tolerated in our study population with minimal adverse effects. Five dogs developed azotemia and although urine culture was negative for bacterial growth in all instances, azotemia resolved with enrofloxacin antibiotic therapy and was therefore suspected to be secondary to pyelonephritis. Severe ureaplasma infections are a known adverse event associated with rituximab treatment in people, some of which are diagnosed with concurrent azotemia and subsequent renal failure.^49^, ^50^, ^51^ In the human cases documented, improvement of clinical signs and azotemia was seen after treatment with fluroquinolone antibiotics.^49^ With these patients, it is hypothesized that rituximab may increase the risk of this infection because of peripheral immunoglobulins loss. As ureaplasma is not detectable via routine urine culture and requires a molecular diagnostic,^51^ it would not have been identified using the standard methodologies applied to dogs treated in this study.

Only 1 dog demonstrated evidence of acquired Type I hypersensitivity after treatment with 1E4‐cIgGB in this study. Infusion reactions are well documented in people treated with rituximab, although these are most common after the initial infusion and decrease with subsequent treatments.^52^, ^53^, ^54^ It is hypothesized that such reactions are because of cytokine release (IL‐6, TNFα, etc.) associated with ADCC and complement mediated B‐cell killing. True hypersensitivity reactions such as type I hypersensitivity (development of anti‐rituximab IgG or IgE antibodies), type III hypersensitivity (manifesting as fever rash), or type IV hypersensitivity (delayed) are also documented in people.^52^, ^53^, ^54^ Importantly, rituximab treatment is discontinued in approximately 10% of people because of infusion or hypersensitivity reactions.^52^

In conclusion, our data demonstrates that the canine specific anti‐CD20 monoclonal antibody 1E4‐cIgGB is effective in depleting B‐cells in dogs with DLBCL. 1E4‐cIgGB is well tolerated with minimal side effects and can be safely administered in combination with doxorubicin chemotherapy and other small molecule inhibitors designed to modulate the immune system (KPT‐9274, RV1001, TAK‐981). Future prospective studies designed to interrogate the utility of 1E4‐cIgGB in combination with single agent doxorubicin and CHOP are warranted to better define its impact on outcomes of dogs with DLBCL.

## CONFLICT OF INTEREST DECLARATION

Authors declare no conflict of interest.

## OFF‐LABEL ANTIMICROBIAL DECLARATION

Authors declare no off‐label use of antimicrobials.

## INSTITUTIONAL ANIMAL CARE AND USE COMMITTEE (IACUC) OR OTHER APPROVAL DECLARATION

Approved by the Tufts University IACUC, G2017‐110 approved 10/19/2017; G2020‐82 approved 8/26/2020.

## HUMAN ETHICS APPROVAL DECLARATION

Authors declare human ethics approval was not needed for this study.

## Supporting information


**Table S1:** Immunotherapeutic agents used in treatment protocols.
**Table S2:** Response by cohort.
**Table S3:** Summary statistics of CD21+ and CD5+ counts over time.
**Table S4:** Summary statistics of CD21+ and CD5+ cells by best response.
**Table S5:** Summary statistics of CD21+ and CD5+ cells by completion of protocol.
