# Supplementary material for: Safety and biologic activity of a canine anti‐CD20 monoclonal antibody in dogs with diffuse large B‐cell lymphoma
Source: J Vet Intern Med. 2024 Apr 25;38(3):1666–74. doi: 10.1111/jvim.17080 (PMC11099711; doi:10.1111/jvim.17080)
Supplement: Supplementary file 1 — Table S1: Immunotherapeutic agents used in treatment protocols. Table S2: Response by cohort. Table S3: Summary statistics of CD21+ and CD5+ counts over time. Table S4: Summary statistics of CD21+ and CD5+ cells by best response. Table S5: Summary statistics of CD21+ and CD5+ cells by completion of protocol. [file JVIM-38-1666-s001.pdf]

Supplemental Table 1. Immunotherapeutic agents used in treatment protocols

Agent	Mechanism of Action	References
**KPT-9274**	KPT-9274 (Karyopharm Therapeutics) is a first-in-class orally bioavailable dual inhibitor of NAMPT and PAK4. It has shown activity against a range of solid and hematological malignancies by reducing growth via modulating PAK4, Wnt/β-catenin signaling, and inhibiting NAD synthesis.	Dittrich K, Yildiz-Altay U, Qutab F, et al. Baseline tumor gene expression signatures correlate with chemoimmunotherapy treatment responsiveness in canine B cell lymphoma. PLoS One 2023;18:e0290428.<br><br>Li Y, Lu Q, Xie C, et al. Recent advances on development of p21-activated kinase 4 inhibitors as anti-tumor agents. Front Pharmacol 2022;13:956220.<br><br>Khan HY, Uddin MH, Balasubramanian SK, et al. PAK4 and NAMPT as Novel Therapeutic Targets in Diffuse Large B-Cell Lymphoma, Follicular Lymphoma, and Mantle Cell Lymphoma. Cancers (Basel) 2021;14.<br><br>Kumar S, Schoonderwoerd MJA, Kroonen JS, et al. Targeting pancreatic cancer by TAK-981: a SUMOylation inhibitor that activates the immune system and blocks cancer cell cycle progression in a preclinical model. Gut 2022;71:2266-2283.<br><br>Mitchell SR, Larkin K, Grieselhuber NR, et al. Selective targeting of NAMPT by KPT-9274 in acute myeloid leukemia. Blood Adv 2019;3:242-255.
**RV1001**	RV1001 (Rhizen Pharmaceuticals) is an orally bioavailable potent and selective PI3Kδ inhibitor with strong hinge binding interaction at Val-882. RV1001 inhibits growth of B-cell and T-cell lymphoma cell lines in a δ-isoform selective manner and exhibits anti-cancer activity in murine xenograft tumor models.	Dittrich K, Yildiz-Altay U, Qutab F, et al. Baseline tumor gene expression signatures correlate with chemoimmunotherapy treatment responsiveness in canine B cell lymphoma. PLoS One 2023;18:e0290428.<br><br>Gardner HL, Rippy SB, Bear MD, et al. Phase I/II evaluation of RV1001, a novel PI3Kδ inhibitor, in spontaneous canine

		lymphoma. PLoS One 2018;13:e0195357.<br><br>London CA, Rippy SB, Bear MD, et al. Abstract 4700: The novel and selective PI3Kδ inhibitor, RV1001, displays single agent biologic activity in spontaneous canine NHL. Cancer Research 2015;75:4700-4700.
**TAK-981**	TAK-981 (Takeda Pharmaceuticals) is a small molecule SUMO-activating enzyme inhibitor that abrogates sumoylated protein-mediated cellular processes including proliferation, DNA repair, metastasis and survival. It also enhances Type 1 IFN-mediated signaling, activating innate effector cells and enhancing anti-tumor immune responses.	Dittrich K, Yildiz-Altay U, Qutab F, et al. Baseline tumor gene expression signatures correlate with chemoimmunotherapy treatment responsiveness in canine B cell lymphoma. PLoS One 2023;18:e0290428.<br><br>Lam V, Roleder C, Liu T, et al. T Cell–intrinsic Immunomodulatory Effects of TAK-981 (Subasumstat), a SUMO-activating Enzyme Inhibitor, in Chronic Lymphocytic Leukemia. Molecular Cancer Therapeutics 2023;22:1040-1051.<br><br>Lightcap ES, Yu P, Grossman S, et al. A small-molecule SUMOylation inhibitor activates antitumor immune responses and potentiates immune therapies in preclinical models. Sci Transl Med 2021;13:eaba7791.<br><br>Kukkula A, Ojala VK, Mendez LM, et al. Therapeutic Potential of Targeting the SUMO Pathway in Cancer. Cancers (Basel) 2021;13.<br><br>Langston SP, Grossman S, England D, et al. Discovery of TAK-981, a First-in-Class Inhibitor of SUMO-Activating Enzyme for the Treatment of Cancer. J Med Chem 2021;64:2501-2520.

Supplemental Table 2. Response by cohort

Cohort	Number	Complete Remission	Partial Remission	Overall Response
All	42	33/42 (78.6%)	9 /42 (21.4%)	42/42 (100%)
2	7	5/7 (71.4%)	2/7 (28.6%)	7/7
3	5	4/5 (80%)	1/5 (20%)	5/5
4	6	5/6 (83.3%)	1/6 (16.7%)	6/6
5	13	11/13 (84.6%)	2/13 (15.4%)	13/13
6	11	8/11 (72.7%)	3/11 (27.3%)	11/11

Supplemental Table 3. Summary statistics of CD21+ and CD5+ counts over time

	**D7**	**D21**	**D84**	**D112**	**D196**
**Fraction of Baseline**					
CD21+	N=42	N=39	N=30	N=24	N=17
Mean (SD)	0.25 (0.59)	0.07 (0.16)	0.51 (1.49)	0.38 (0.74)	1.17 (2.02)
Median (Q1, Q3)	0.04 (0.01,0.2)	0.01 (0,0.04)	0 (0,0.14)	0.14 (0.02,0.28)	0.3 (0.17,0.66)
Min, Max	0,3.49	0,0.85	0,6.02	0,2.66	0.01, 7.15
**CD5+**	N=41	N=39	N=29	N=24	N=17
Mean (SD)	1.02 (0.49)	0.96 (0.51)	0.79 (0.45)	0.85 (0.45)	1.17 (0.57)
Median (Q1, Q3)	1.06 (0.71,1.24)	0.79 (0.6,1.39)	0.68 (0.52,0.87)	0.81 (0.52,1.03)	1.15 (0.72,1.65)
Min, Max	0.23, 2.28	0.23, 2.15	0.29, 2.11	0.22, 1.9	0.27, 2.12
**Complete Data Through Day 21**					
**CD21+**	N=39	N=39	n/a	n/a	n/a
Mean (SD)	0.23 (0.59)	0.07 (0.16)			
Median (Q1, Q3)	0.04 (0.01,0.2)	0.01 (0,0.04)			
Min, Max	0, 3.49	0, 0.85			
	p<0.01	p<0.01			
**CD5+**	N=39	N=39	n/a	n/a	n/a
Mean (SD)	1.01 (0.5)	0.96 (0.51)			
Median (Q1, Q3)	1.05 (0.54,1.24)	0.79 (0.6,1.39)			
Min, Max	0.23, 2.28	0.23, 2.15			
	p=0.88	p=0.42			
**Complete Data Through Day 196**					
**CD21+**	N=17	N=17	N=17	N=17	N=17
Mean (SD)	0.37 (0.86)	0.12 (0.22)	0.18 (0.35)	0.42 (0.83)	1.17 (2.02)
Median (Q1, Q3)	0.05 (0.01,0.19)	0.04 (0,0.17)	0 (0,0.14)	0.15 (0.03,0.28)	0.3 (0.17,0.66)
Min, Max	0, 3.49	0, 0.85	0, 1.15	0, 2.66	0.01, 7.15
	p<0.01	p<0.01	p<0.01	p=0.04	p=0.21
**CD5+**	N=17	N=17	N=17	N=17	N=17
Mean (SD)	1.1 (0.45)	1.1 (0.55)	0.9 (0.53)	0.94 (0.48)	1.17 (0.57)
Median (Q1, Q3)	1.06 (0.84,1.29)	1.06 (0.73,1.39)	0.83 (0.57,1.04)	0.84 (0.64,1.22)	1.15 (0.72,1.65)
Min, Max	0.39, 1.9	0.26, 2.15	0.29, 2.11	0.28, 1.9	0.27, 2.12
	p=0.40	p=0.71	p=0.21	p=0.58	p=0.26

Supplemental Table 4. Summary statistics of CD21+ and CD5+ cells by best response

Fraction of Baseline	Complete Remission	Partial Remission
**CD21+**	N=33	N=9
Mean (SD)	0.26 (0.64)	0.2 (0.41)
Median (Q1, Q3)	0.04 (0.01,0.19)	0.02 (0,0.21)
Min, Max	0, 3.49	0, 1.26
**CD5+**	N=32	N=9
Mean (SD)	1.02 (0.54)	1.04 (0.27)
Median (Q1, Q3)	1.03 (0.5,1.26)	1.15 (0.88,1.21)
Min, Max	0.23, 2.28	0.54, 1.32

Supplemental Table 5. Summary statistics of CD21+ and CD5+ cells by completion of protocol

Fraction of Baseline	Completed Protocol	Did Not Complete Protocol
**D7: CD21+**	N=29	N=13
Mean (SD)	0.27 (0.68)	0.2 (0.35)
Median (Q1, Q3)	0.04 (0.01,0.17)	0.02 (0,0.21)
Min, Max	0, 3.49	0, 1.26
**D7: CD5+**	N=29	N=13
Mean (SD)	0.92 (0.46)	1.24 (0.49)
Median (Q1, Q3)	0.88 (0.5,1.15)	1.15 (1.05,1.31)
Min, Max	0.23, 1.9	0.54, 2.28
**D21: CD21+**	N=29	N=10
Mean (SD)	0.08 (0.17)	0.05 (0.11)
Median (Q1, Q3)	0.01 (0,0.04)	0 (0,0.04)
Min, Max	0, 0.85	0, 0.37
**D21: CD5+**	N=29	N=10
Mean (SD)	0.92 (0.52)	1.07 (0.47)
Median (Q1, Q3)	0.76 (0.57,1.15)	0.84 (0.75,1.54)
Min, Max	0.23, 2.15	0.39, 1.82
